# Human milk oligosaccharide composition is affected by season and parity and associates with infant gut microbiota in a birth mode dependent manner in a Finnish birth cohort

**DOI:** 10.1016/j.ebiom.2024.105182

**Published:** 2024-06-04

**Authors:** Dollwin Matharu, Alise J. Ponsero, Marton Lengyel, Agnes Meszaros-Matwiejuk, Kaija-Leena Kolho, Willem M. de Vos, Dora Molnar-Gabor, Anne Salonen

**Affiliations:** a Human Microbiome Research Program, Faculty of Medicine, University of Helsinki, Helsinki, Finland; b DSM-Firmenich, (Formerly: Glycom A/S), Hørsholm, Denmark; c Children's Hospital, University of Helsinki and HUS, Helsinki, Finland; d Laboratory of Microbiology, Wageningen University, the Netherlands

**Keywords:** HMOs, Maternal factors, Early life exposures, *FUT2*, Glycoside hydrolases, Child health

## Abstract

**Background:**

Human milk oligosaccharides (HMOs), their determinants, infant gut microbiota and health are under extensive research; however, seldom jointly addressed. Leveraging data from the HELMi birth cohort, we investigated them collectively, considering maternal and infant secretor status.

**Methods:**

HMO composition in breastmilk collected 3 months postpartum (n = 350 mothers) was profiled using high-performance liquid chromatography. Infant gut microbiota taxonomic and functional development was studied at 3, 6, and 12 months (n = 823 stool samples) via shotgun metagenomic sequencing, focusing on HMO metabolism via glycoside hydrolase (GH) analysis. Maternal and infant secretor statuses were identified through phenotyping and genotyping, respectively. Child health, emphasizing allergies and antibiotics as proxies for infectious diseases, was recorded until 2 years.

**Findings:**

Mother's parity, irritable bowel syndrome, gestational diabetes, and season of milk collection associated with HMO composition. Neither maternal nor infant secretor status associated with infant gut microbiota, except for a few taxa linked to individual HMOs. Analysis stratified for birth mode revealed distinct patterns between the infant gut microbiota and HMOs. Child health parameters were not associated to infant or maternal secretor status.

**Interpretation:**

This comprehensive exploration unveils intricate links between secretor genotype, maternal factors, HMO composition, infant microbiota, and child health. Understanding these nuanced relationships is paramount for refining strategies to optimize early life nutrition and its enduring impact on long-term health.

**Funding:**

Sweet Crosstalk EU H2020 MSCA ITN, 10.13039/501100002341Academy of Finland, Mary and Georg C. Ehrnrooth Foundation, 10.13039/501100004212Päivikki and Sakari Sohlberg Foundation, and Tekes.

**Research in context**

**Evidence before this study**

Early life health modulators, such as maternal factors, breastfeeding, human milk oligosaccharides (HMOs), fucosyltransferase 2 *(FUT2)* secretor genotype and gut microbiota have garnered growing interest. Existing studies are often small and lack background information, hindering granular analysis. The combined effect of maternal and infant secretor status is rarely explored, limiting comprehensive insights.

**Added value of this study**

Our study based on healthy term infants from the HELMi birth cohort explores the relationships between HMO profiles in maternal milk and various factors, such as maternal characteristics, infant and maternal secretor status, infant gut microbiota development until the age of one, and health outcomes up to age two. Notably, we identified parity, seasonality, and maternal health influencing the HMO profiles. Importantly, no significant impact on children's microbiota or health outcomes was observed based on either maternal or infant secretor status.

**Implications of all the available evidence**

Maternal and background exposures significantly influence HMO profiles, which further influence infant microbiota development, more prominently in a birth mode-specific manner. While previous studies identified effects of maternal secretor status on infant gut microbiota, they often had limited sample sizes and short follow-up periods. Our findings call into question the existing notion that the non-secretor milk is inferior. We show that both *FUT2*-independent and -dependent HMOs sufficiently support infant microbiota and health. This study is crucial for those interested in early life nutrition, emphasizing the importance of breastfeeding for infant health regardless of secretor status.

## Introduction

Breastfeeding plays a pivotal role in influencing infant health and developmental outcomes.^1^ Human milk oligosaccharides (HMOs) have gained remarkable interest in the recent years due to their biological implications.^2^ These structurally diverse glycans help enhance the intestinal barrier function, modulate intestinal epithelial cells, and have been shown to have immunomodulatory and antiviral properties.^3^ HMOs are present only in human breast milk and constitute the third largest solid component in mothers' milk after lactose and lipids, being indigestible to the host but can be specifically metabolized by certain bacteria in the infant gut.^4^ HMO concentrations peak in colostrum and gradually decline postpartum.^5^ Importantly, two genes fucosyltransferase 2 and 3 (*FUT2* and *FUT3*), define the secretor and the Lewis genotypes, respectively, that contribute to a large proportion of the HMO compositional variability in mothers’ milk.^6^ *FUT2* and *FUT3* influence fucosylation in human milk, other bodily secretions and mucosal tissues.^7^ Fucose provides an endogenous source of nutrients and attachment sites for the colonizing microbes.^8^ The *FUT2 a*nd *FUT3 g*enes form the genetic basis of the four milk group types, milk group 1 (*FUT2+, FUT3+*), milk group 2 (*FUT2−, FUT3+*), milk group 3 (*FUT2+, FUT3−*) and milk group 4 (*FUT2−, FUT3−*).^9^ Besides genetic factors, other maternal and environmental exposures such as dietary factors have been suggested to influence HMO concentrations.^10^^,^^11^ An emerging body of evidence indicates potential influences from parameters such as delivery mode, parity, maternal age, geographic location, and seasonal variations, on the HMO profiles in breast milk.^11^ While still in early stages, these findings imply complex yet consistent associations between multifaceted maternal and environmental factors and the HMO profiles.

Early life microbiota plays an important role in health and development during infancy and later in life.^12^ Several factors shape the early life gut microbiota, including birth mode, infant nutrition, environmental exposures, and genotype, among others.^12^, ^13^, ^14^ In particular, dietary factors have a profound influence on the initial microbiota development, often extending to long-term health outcomes.^15^ The exposure to HMOs is pivotal in supporting the growth of specific beneficial early colonizers in the infant gut, notably from the genus *Bifidobacterium*.^16^ Certain species, such *as B. bifidum, B. breve, and B. longum subsp. infantis*, exhibit unique mechanisms to interact with and metabolize different HMOs as their sole carbon source.^17^ Among the early gut colonizers, *Bacteroides* spp. can also utilize HMOs,^18^^,^^19^ at least *B. fragilis, B.vulgatus, B. caccae* and *B. thetaiotaomicron* being able to grow solely on HMOs.^20^ Emerging evidence suggests there could also be other HMO utilizers in the infant gut besides *Bifidobacterium* and *Bacteroides,* such as *Akkermansia*.^21^ Cohort studies that record infant microbiota profiles and HMO composition hold paramount importance in unraveling their intricate interplay and their potential influence on child health. Several studies have investigated the relationship between HMOs and infant gut microbiota, most of them on sample sizes <100 and using 16S rRNA gene sequencing approach, providing limited resolution on taxonomic profiles and only imputed functional information.^22^ A recent report by Derrien et al. showed the relationship between HMOs and microbiota using shotgun data in 90 Kenyan infants with samples spread over a window of 5 months (6–11-month-old infants), after introducing solid foods.^23^ A cross-sectional study by Barnett et al. investigated the associations between HMOs in exclusively breastfed 220 Dutch infants and their microbiota profiles at 1 month using 16S rRNA gene amplicon sequencing approach.^24^

Here, we leverage the Finnish Health and Early Life Microbiota (HELMi) birth cohort^25^ to explore the interplay between early life factors, HMO composition, infant gut microbiome, and child health. We hypothesized that (1) maternal and early life factors associate with the HMO composition and concentration in mothers' milk, (2) the *FUT2* secretor status of both the mother and the infant contribute to infant gut microbiota composition, (3) the HMO profiles along with early life factors shape the bacterial composition and functional potential of the infant gut microbiome, and (4) the HMO profiles associate to specific infant health outcomes. To address these questions, we generated HMO profiles from breast milk samples (n = 350 mothers) collected three months after childbirth. The infant fecal microbiota composition was assessed by shotgun metagenomic sequencing at 3 months, 6 months and 1 year. Utilizing the comprehensive metadata collected in the HELMi cohort, we addressed associations between the HMO profiles, and maternal characteristics and perinatal factors, as well as infant health outcomes up to the age of 2 years. Alongside maternal secretor status, determined by the presence or absence of 2'fucosyllactose (2′FL) in breast milk, we also determined the infant secretor status using *FUT2* genotyping, to study the effect of both mother's and infant's secretor status on infant gut microbiota. To the best of our knowledge this is the largest study of its kind that includes maternal milk HMO composition and longitudinal infant gut microbiota samples, along with comprehensive metadata including child health outcomes. Also, most previous studies investigating the effect of maternal secretor status on infant microbiota and health have rarely included infant secretor status too. These approaches allowed us to delve deeply into the interactions between maternal and perinatal factors, HMO concentrations in breast milk, maternal and infant secretor status, infant gut microbiome, and infant health.

## Methods

### Cohort and collection of the fecal and breast milk samples

This study includes samples from the broader Health and Early Life Microbiome (HELMi) birth cohort (NCT03996304), which includes 1055 Finnish infants followed from birth to 2 years of age.^25^ Infants included in the HELMi cohort were mainly born in the Uusimaa region in 2016–18, with a birth weight of >2.5 kg, without known congenital defects, and born between the gestational weeks 37 and 42. The infants were healthy, except three infants had heart-related diagnosis (coarctation of the aorta, supraventricular takycardia, and ventricular septal defect), and one Incontentia pigmentis. No other long-term disease (with the exception of allergic diseases) was diagnosed for these infants before 3 months. In this study, a subset of 374 families from the cohort was selected based on the following criteria: availability of breast milk sample, reported continuous breastfeeding at least until 13 weeks post-partum, availability of corresponding infant fecal samples, and completeness of the online questionnaire data. However, a total of 24 milk samples were excluded due to insufficient breastmilk sample quantity, leaving a final number of 350 breastmilk samples included in this study.

Breastmilk samples were collected at 3 months post-partum, with the assistance of a study nurse at the study visit. Mothers were provided with detailed instructions on collecting breastmilk samples. Samples were expressed wearing gloves, from the breast that was not used to feed the infant for at least 1 h prior to milk expression. Samples were stored at −20 °C for a maximum of one month, brought to the laboratory in frozen form and stored at −80 °C until processing.

Fecal samples from infants were collected to characterize the microbiota composition. This study included infant fecal samples collected at 3 months, 6 months, and 1 year. Parents collected the infant fecal samples at home and stored them in −20 °C freezers (up to 6 months), until shipment to the laboratory where the samples were stored in −80 °C until processing. At three months of age, 20 parents reported the child to have a minor illness (common cold) at the time of collection. Records on early life exposures were collected via online questionnaires that parents answered, as well as hospital records.^26^ Missing or impossible values for each variable were excluded from each individual models, and the number of missing values for each variable is listed in Supplemental File 1.

### HMO extraction and quantification

An aliquot of 200 μl of thawed breast milk was first centrifuged at 5000 g for 15 min, followed by mixing 100 μl of the aqueous phase with 900 μl of a 1:1 (V/V) mix of acetonitrile and water to precipitate dissolved proteins. This was followed by another centrifugation step at 12,000 g for 5 min, then 500 μl of the supernatant was concentrated using an Amicon Centrifugal Filtration Unit (3 kDa nominal molecular weight cutoff, Merck) and centrifuged at 12,000 g for 30 min. All centrifugations were performed at room temperature. The filtrate was used for further analysis.

HMOs were derivatized by reductive amination approach using benzocaine and picoline borane, panose was used as an internal standard and afterwards analyzed using High-performance liquid chromatography-ultraviolet (HPLC-UV) equipped with an amide column (Thermo Fisher Scientific Accucore Amide–Hydrophilic Interaction Liquid Chromatography, 2.6 μm particle size, 2.1 × 150 mm). A multi-step ternary gradient of acetonitrile, Milli-Q water, and 10 mM ammonium formate—formic acid buffer (pH 3.0) was used. Fourteen HMOs were analysed (12 as individual entities and two as unresolved moieties): 2′-fucosyllactose (2′FL), 3-Fucosyllactose (3FL), Lacto-N-fucopentaose I (LNFP1), Lacto-N-difucohexaose I (LNDFH1), Lacto-N-fucopentaose V (LNFP5), Lacto-N-neotetraose (LNnT), Lacto-N-tetraose (LNT), Sialyl-lacto-N-tetraose c (LSTc), Sialyl-lacto-N-tetraose b (LSTb), 3′-Sialyllactose (3′SL), 6′-Sialyllactose (6′SL), Disialyllacto-N-tetraose (DSLNT), Lacto-N-fucopentaose II + III (LNFP2 + 3), and Lacto-N-difucohexaose II + III (LNDFH2 + 3). The calibration solution contained eight HMOs (2′FL, 3FL, DFL, 3′SL, 6′SL, LNT, LNnT, and LNFP1). Of these, DFL could not be measured selectively, but the rest were separated from each other. In addition to these, the sum of LNFP2 and LNFP3, LNFP5, LNDFH1, the sum of LNDFH2 and LNDFH3, LST-b, LST-c, and DSLNT were quantified, using the LNFP1 calibration curve with a response factor calculated from the respective molar masses of the compounds, since oligosaccharides have identical response on a molar basis as a consequence of the labeling technique. Chromeleon 7.2.10 was used for data processing.

### Determination of secretor status and milk groups

Milk samples were classified as either secretors (Se+) or non-secretors (Se−), based on the presence or absence of 2′ fucosyllactose (2′FL) and Lacto-N-Fucopentose 1 (LNFP1), and Lewis + (Le+) or Lewis—(Le−) based on presence or absence of Lacto-N-difucohexaose II (LNDFH2) (Supplemental Table S1). The samples were further divided into four milk groups based on the presence or absence of specific HMOs, as described by Tonon et al.^6^ Those that were secretors (Se+) were placed in Milk Group 1 or 3 and non-secretors (Se−) in Milk groups 2 or 4. In Milk groups 1 and 3 subgrouping was based on two HMOs: LNFP1 and LNDFH1. Samples that had both HMOs were assigned to Milk group 1, while those that did not have LNDFH1 were put in Milk group 3. While both Milk group 1 and 3 are Se+, Milk group 1 is Le+, but Milk group 3 is Le−. Those samples that were Se−, i.e., depleted in both 2′FL and LNFP1, were also classified further into two milk groups: Milk Group 2 and Milk Group 4. All samples in both Milk Group 2 and 4 lack 2′FL, LNFP1, and LNDFH1, but the ones in Milk Group 2 have LNDFH2, while those falling in Milk Group 4 lack it. While both Milk group 2 and 4 are Se−, milk group 2 is Le+, while milk group 4 is Le−. Furthermore, all samples always had 3FL. Spearman's correlation coefficients between the HMO concentrations were determined using the R package ggcorrplot (0.1.4). For determination of infant secretor status, buccal swabs were collected at age of 3 months and used for DNA extraction using Investigator Lyse & Spin Basket Kit and QIAsymphony SP system with Buccal Extraction Protocol (Qiagen, Valencia, CA). *FUT2* genotype was determined by genotyping the FUT2 single nucleotide polymorphism rs601338 as described.^27^ As infant's secretor status is determined by a combination of *FUT2* alleles from both parents, in mother–infant pairs all four combinations are possible (both secretors, both non-secretors, discordant either way).

### Shotgun metagenomic sequencing, taxonomic and functional profiling

Fecal DNA was extracted using repeated bead-beating (RBB) method. Fecal material (250–340 mg) was suspended in 0.5–1 ml of sterile ice-cold PBS, and 250 μl of the fecal suspension was mixed with 340 μl of RBB lysis buffer (500 mM NaCl, 50 mM Tris–HCl (pH 8), 50 mM EDTA and 4% SDS) in a bead-beating tube from Ambion Magmax™ Total Nucleic Acid Isolation Kit (Life Technologies, Carlsbad, CA, United States). After repeat bead-beating, 200 μl of the supernatant was used for DNA extraction with a KingFisherTM Flex automated purification system (Thermo Fisher Scientific, Waltham, MA, United States) using MagMAXTM Pathogen High Vol. DNA was quantified using the Quanti-iT™ Pico Green dsDNA Assay (Invitrogen, San Diego, CA, United States). Metagenomic sequencing was carried out using MGI technology. Library preparation and circularisation of equimolarly pooled libraries was carried out with MGIEasy FS DNA Library Prep Set (MGI Tech, Shenzhen, China) and sequenced using DNBSEQ-G400RS High-throughput Sequencing Set (FCL PE150) according to manufacturer's instructions (MGI Tech, Shenzhen, China). Sequencing reads' quality control (QC) was performed using Fastqc (v 0.12.1) and trimGalore (v 0.6.4),^28^ and human reads were filtered using Bowtie2 (v 0.6.5) against the GRCH build 38, patch release 14 (available from https://https.ncbi.nlm.nih.gov/grc/human). After QC and human read filtering, metagenomes with less than 20 million paired-end reads were excluded. Taxonomic profiling of the bacterial communities was obtained using Kraken2 (v 2.1.3)^29^ and Braken (v 2.7)^30^ against the Humgut database^31^ (available from https://arken.nmbu.no/∼larssn/humgut/). Functional profiles were obtained using HumaNn 3.0 (v 3.1.8)^32^ against the UniRef90 database. Gene family counts were normalized as gene copy number per million reads and mapped to MetaCyc pathway definitions. Carbohydrate-active enzymes (CAZy) were assessed with DbCan. Briefly, QC reads were assembled using Megahit (v 1.2.9),^33^ and proteins were predicted using Prodigal (v 2.6.3)^34^ and dereplicated using CD-Hit (v 4.8.1)^35^ (95% protein identity). Protein clusters were annotated into CaZy families and sub-families using DbCan (v 4.0.0)^36^ and reads were mapped against the clusters using Bowtie2 (v 2.4.1).^37^ The predicted substrates for each CaZy sub-families were obtained from DbCan v4. Counts for CAZy sub-families of interest were normalized using gene copy per million reads.

### Associations between HMO concentrations, technical and maternal variables

The associations between technical, maternal factors and HMO concentrations in breastmilk were assessed using generalized linear mixed models on the HMO concentration z-scores, using the Multivariate Association with Linear Models package (MaAsLin2 v 1.7.3).^38^ Any association with a false discovery rate (FDR)- adjusted q-value <0.05 was considered significant. In this analysis, milk groups were included as fixed effects in the models.

The permutational analysis of variance (PERMANOVA) was performed in R using the vegan package^39^ function adonis2 (v 2.6–4) using a Euclidean distance computed on the z-scores transformed HMO concentrations.

### Association between infant gut microbiota and HMO concentrations

Fecal community types were identified using hierarchical clustering performed on the bacterial taxonomic profiles aggregated at the family level using the function hclust from the vegan package (v 2.6–4), using Wald.D2 method on Bray–Curtis distance. The appropriate number of clusters was determined by silhouette analysis and the clusters were confirmed by PERMANOVA at family level using Aitchison and Bray–Curtis distance.

The associations between overall microbiota composition and perinatal and maternal variables such as milk groups or secretor status, as well as background exposures, were studied using PERMANOVA on both Bray–Curtis and Aitchinson distances. The list of the variables tested is provided in Supplemental File 1. The models included sequencing depth and the significant biological covariates (birth mode, intra-partum antibiotics (IA) and parity).

The associations between secretor statuses or individual HMOs and the relative abundance of bacterial taxa at species level, or with CAZy annotations were assessed using generalized linear mixed models from MaAsLin2. HMO concentrations were transformed into z-scores, and models included birth mode, IA and parity as fixed effects. Alternatively, independent models were run for each birth modality, including parity as fixed effects. Any association with an FDR-adjusted q-value <0.15 was considered significant.

### Association between infant health at 1 and 2 year and maternal secretor status

Several infant phenotypic outcomes were recorded, such as diagnoses of allergic diseases, antibiotic use as proxy of infectious diseases, and growth trajectory up till 2 years of age. The list of the variables analyzed with respect to infant health outcomes is provided in Supplemental File 1. The associations between HMO concentrations and infant phenotypic outcomes were assessed using generalized linear mixed models from MaAsLin2, and any association with FDR-adjusted q-value <0.15 was considered significant.

### Ethics

The cohort study was conducted in accordance with the principles of the Helsinki Declaration and was approved by the ethical committee of the Hospital District of Helsinki and Uusimaa. Upon enrolment, the parents signed an informed consent.

### Role of funders

The funders played no role in the study design, data collection, data analysis, data interpretation, and paper writing.

## Results

### Characteristics of the cohort, HMO profiles in mothers’ milk and infant gut microbiota

**Fig. 1:**
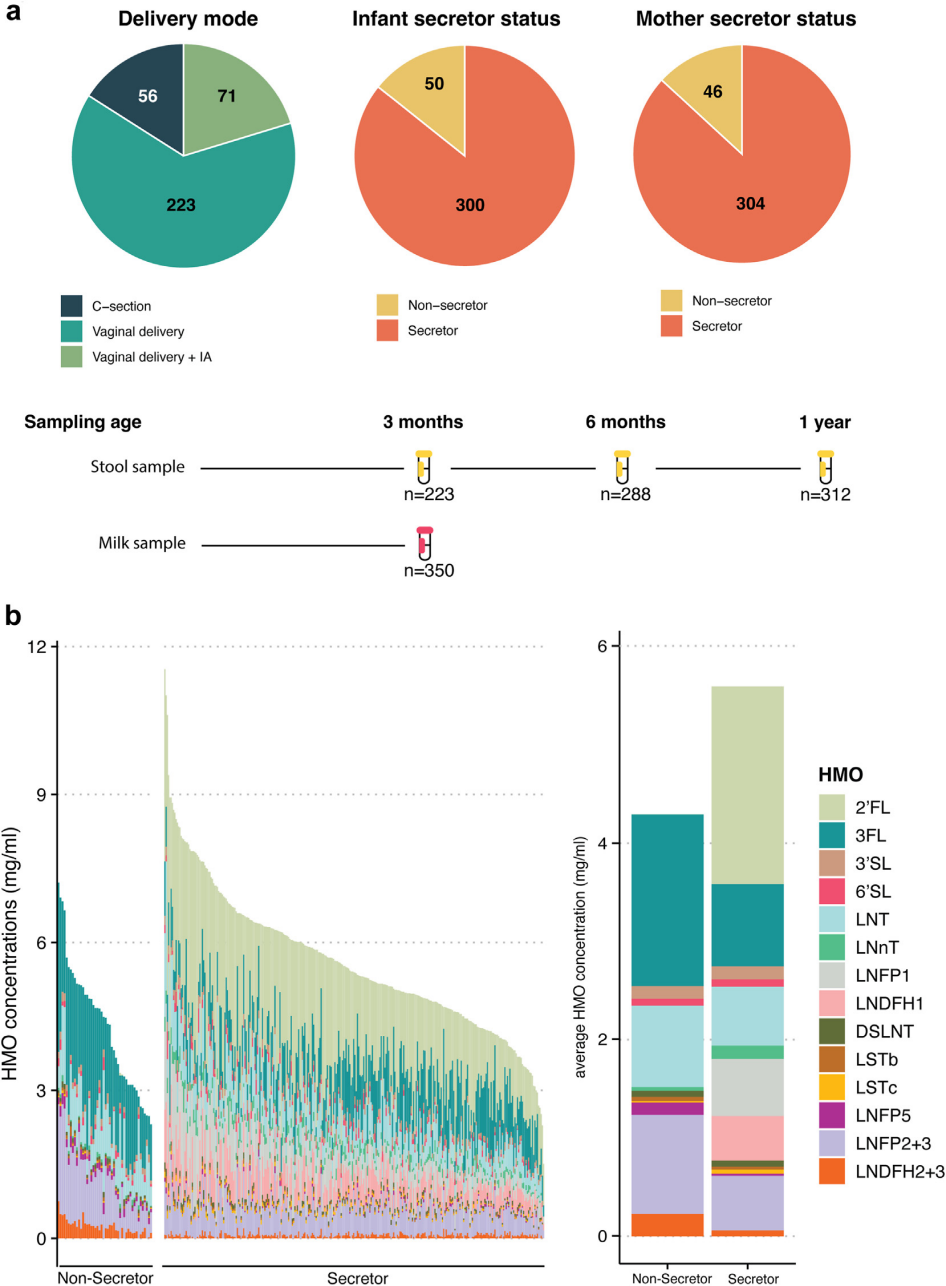
**Cohort overview and study design** (a) scheme of the sampling strategy and cohort characteristics. (b) Human milk oligosaccharide (HMO) composition and concentrations in mothers' milk, showing the absolute HMO composition across 350 breast milk samples (panel 1) and the average concentrations of HMOs by secretor status (panel 2) expressed in mg/ml.

From the 350 families included in our study, 56 (16%) children were born by caesarean delivery and the rest, 294 (84%), were delivered vaginally. All the mothers with caesarean delivery and 71 mothers who delivered vaginally, were given intrapartum antibiotics (IA). All families included in this study reported continuous breastfeeding at least until 13 weeks post-partum. Until the age of 3 months, 99/350 (28.2%) had reported one or more instances of formula feeding, in addition to breastfeeding. At the age of 6 months, 335 (95.7%) families, and at 1 year, 279 (79.7%) families reported breastfeeding in addition to solid foods. The median age of starting solid food was 22 weeks (Interquartile range (IQR) = 19–25). Shotgun metagenomic microbiome profiles from infant stool samples were available at the age of 3 months (n = 223), 6 months (n = 288), 1 year (n = 312), for the assessment of fecal microbiota composition and functions. Fig. 1a provides an overview of the cohort characteristics and study design.

The cohort's baseline characteristics stratified by maternal secretor status are summarized in Supplemental Table S2. Based on the presence and absence of 2′FL in breast milk, 304 (86.9%) mothers were secretors and 46 (13.1%) were non-secretors. Milk from non-secretor mothers contained significantly less total HMOs than the milk of secretor mothers (Wilcoxon test *p*-value <.05). The median total HMO concentration in the secretor milk was 5.4 mg/ml (IQR = 4.55–6.25), and 4.5 mg/ml in non-secretors’ milk (IQR = 3.5–5.5) (Fig. 1b). We assessed the correlations between HMOs across the dataset and stratified by secretor status (Supplemental Figure S1). As expected, *FUT2* dependent HMOs like 2′FL and LNFP1 exhibited strong positive correlations (Spearman correlation, rho >0.7), as did 3FL and LNDH2 + 3. In contrast, 3FL and LNFP1 displayed a strong negative correlation (Spearman correlation, rho = −0.7) both across the whole dataset and in secretor milk. In non-secretor milk, the concentrations of LNDFH2 + 3 and LNFP2 + 3, LNDFH2 + 3 and 3FL, and LSTb and LNFP5 were strongly positively correlated (Spearman correlation, rho >0.7).

Several HMO concentrations, such as 3FL, LNFP5, LNFP2 + 3, LNDFH2 + 3, LNT and LSTb, were significantly higher in the milk of non-secretor mothers (Wilcoxon test, q-value <0.05), while several others including total fucosylated, total HMOs, particularly LNnT and LSTc were higher in secretor mothers. On the other hand, total neutral, total sialylated, specifically 3′SL, 6′SL and DS-LNT concentrations did not differ between the secretor groups (Supplemental Figure S2). The milk samples were classified into the 4 human milk groups, as determined by the presence or absence of specific HMOs (see methods). In this study, 269 (76.9%) mothers belonged to Milk Group 1 (Se+, Le+), 40 (11.4%) to Milk Group 2 (Se−, Le+), 35 (10%) to Milk Group 3 (Se+, Le−), and 6 (1.7%) to Milk Group 4 (Se−, Le−) (Supplemental Figure S3).

In our dataset, 50 (14.3%) infants were non-secretors while 300 (85.7%) infants were secretors. The infant microbiota composition was assessed using shotgun metagenomic sequencing at 3 months, 6 months and 1 year. As expected, we observed an increase in alpha diversity and a decrease in inter-individual beta diversity with age (data not shown). At 3 months of age (n = 233), the most abundant bacterial families had the following average relative abundances: *Bacteroidaceae* (16.9%), *Bifidobacteriaceae* (14.5%), *Lachnospiraceae* (12.5%), *Enterobacteriaceae* (9.4%), and *Oscillospiraceae* (7.3%). The microbial communities clustered into three fecal community types (FCT) using hierarchical clustering on the taxa profiles at the family level. Each community type was dominated by distinct taxa. FCT1 (n = 88 infants) was dominated by *Enterobacteriaceae*, FCT2 (n = 56 infants) was dominated by *Bifidobacteriaceae* and FCT3 (n = 79 infants) was dominated by *Bacteroidaceae* (Supplemental Figure S4A). FCT1 displayed a significantly lower alpha-diversity than FCT 2 and FCT 3 (Shannon diversity metric, Wilcoxon test *p* < 0.001) (Supplemental Figure S4B). These FCT captured 43% of the total taxonomic variance of the samples (PERMANOVA on Bray–Curtis distances on family level, *p* < 0.05, permutations = 999, model adjusted for sequencing depth), and showed an expected strong association with the mode of delivery, with 78% of infants being caesarean delivered within the FCT1 (Fisher's exact test, *p* < 0.05), while maternal IA was not associated with the FCTs (Fisher's exact test, *p* > 0.05 on vaginally delivered infants). Birth mode, IA and parity were found to have a significant association with the infant microbiota variation (PERMANOVA Bray–Curtis distance on family level, *p* < 0.05, permutations = 999, model adjusted for sequencing depth) and were therefore chosen as biological covariates in subsequent models.

### Season of sampling affects the HMO composition in breastmilk

**Fig. 2:**
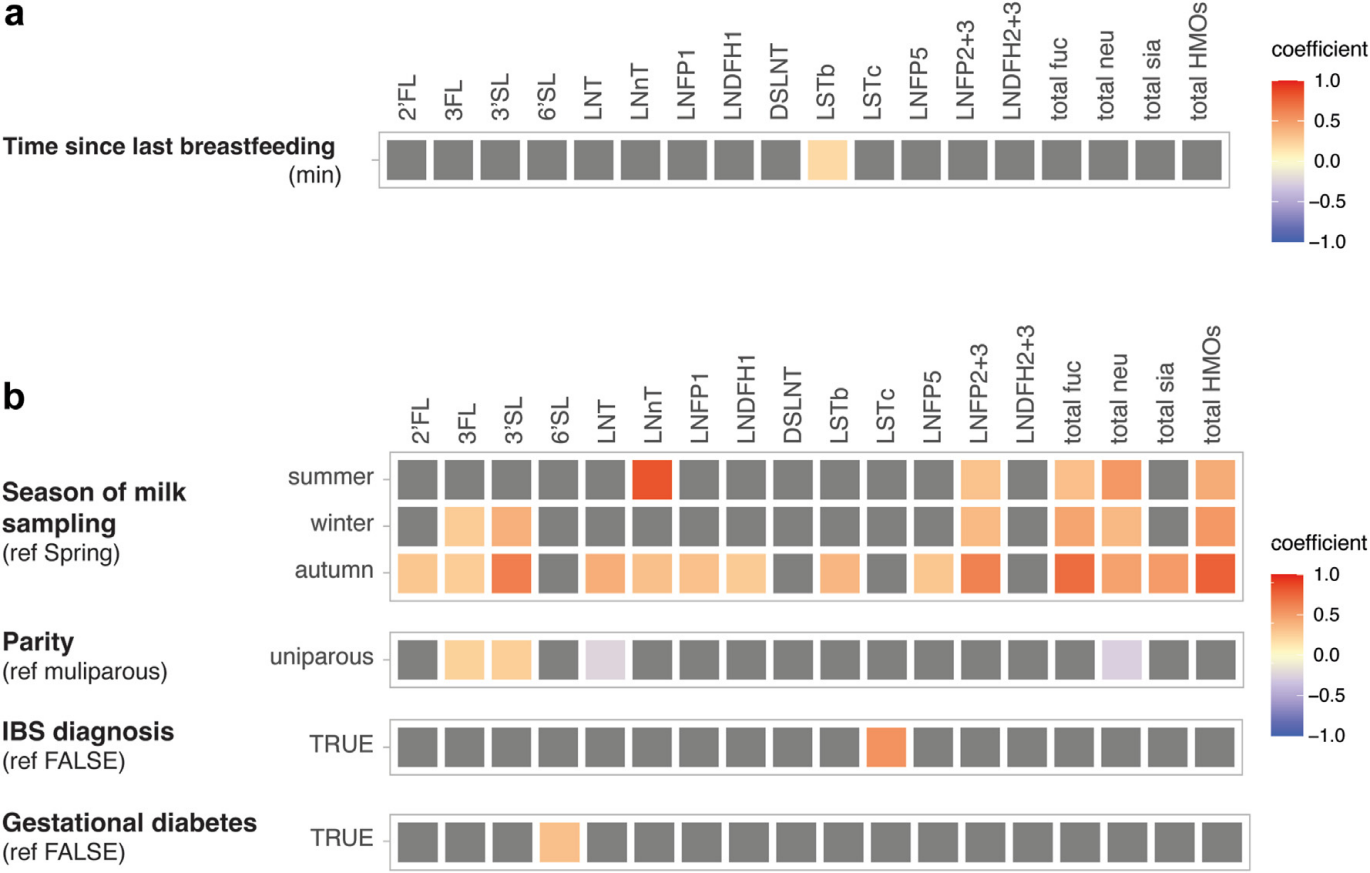
**Association between technical and maternal factors with HMO composition**. Association between (a) technical factors, (b) maternal factors and HMO compositions. Coefficients of association were obtained from linear models on the z-score transformed HMO concentrations, with the milk group included as fixed effect in the models. Associations with FDR adjusted q-values <0.05 were considered statistically significant, with all original unadjusted *p*-values <0.05. Tile coloring reflects direction and magnitude of coefficient, grey tile represents non-significant association. The other technical and maternal variables considered in this analysis, that showed no significant associations with HMOs concentrations were not plotted (complete list in Supplemental File 1).

First, we assessed the impact of technical variables on the measured HMO concentrations to address potential confounding bias. We investigated the potential impact of the time between the last breastfeeding and sample collection, the time of the day (morning or afternoon) when sample collection took place, and the sampling date. Importantly, these investigated technical variables had no or limited significant impact on the measured HMO concentrations (Linear mixed effect models, q < 0.05) (Fig. 2a). Next, we assessed the association between the HMO composition in breastmilk and maternal factors including maternal lifestyle and exposure variables (physical activity level, fatty and folic acid supplementation during pregnancy, season of milk collection, and medications during breastfeeding), age, and health-related variables (diagnosed food allergy, asthma, atopy, diagnosed irritable bowel syndrome (IBS), maternal body mass index (BMI) before pregnancy), and delivery related variables (parity, delivery mode, pregnancy duration and gestational diabetes). The list and definition of all variables included in this study is available in Supplemental File 1.

The number of milk samples collected were highest in autumn (n = 96 samples) and lowest in spring (n = 74 samples), and season was found to be significantly associated with several HMO concentrations (Fig. 2b), as well as the total HMO concentrations (Linear mixed effect models, q < 0.05). Additionally, parity and maternal diagnosis for IBS (n = 23) were associated to significant changes in HMO concentrations- 3FL, 3′SL, LNT and total neutral HMOs with parity, and LSTc with maternal IBS, while gestational diabetes was associated to 6′SL (Fig. 2b; linear mixed effect models, q < 0.05). To assess the total cumulative variation within the HMOs explained by the variables investigated in this study, a PERMANOVA cumulative model was constructed with the identified technical and biological variables: Milk group, length of sample storage, season of milk collection and parity. The total variation explained by this model reached 30%, with the milk group explaining 27% of the variance, the season of milk collection explaining 2%, and parity explaining less than 1% variation.

### HMO composition, but not secretor status, was associated with the infant gut microbiota composition and functions at 3 months

**Fig. 3:**
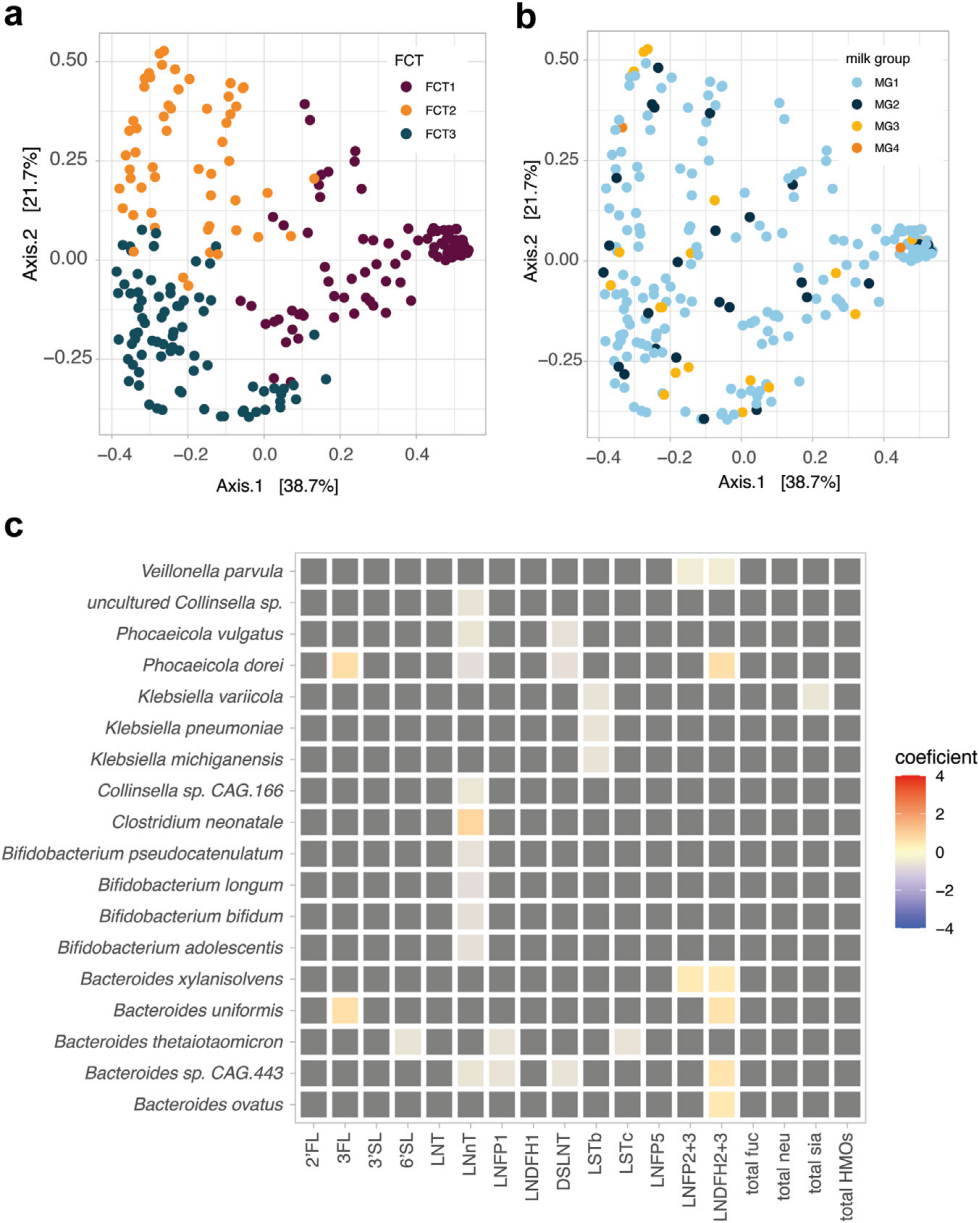
**Association between HMO composition and infant gut microbiota taxonomic composition at 3 months**. Overview of the infant gut microbiota composition at 3 months colored by (a) FCT or (b) milk groups. PCoA on the species-level taxonomic composition of the fecal samples using Bray–Curtis distance. (c) Association between HMO compositions and the infant gut microbiota at 3 months. Coefficients of association were obtained from linear models on the log transformed species-level taxonomic composition, models including parity, birth mode and IA (maternal intrapartum antibiotics) as fixed effects. Only species reaching a minimum relative abundance of 1% in 10% of the samples are shown. The associations with FDR adjusted q-values <0.15 were considered statistically significant, with original unadjusted *p*-values <0.05. Tile coloring reflects direction and magnitude of coefficient, grey tile represents non-significant association.

We studied the associations between maternal secretor status, infant secretor status, and the infant gut microbiota at 3 months (n = 223). Neither maternal nor infant's secretor status had significant associations with the infant microbiota composition (Supplemental Figure S5A–D) (PERMANOVA, Bray–Curtis distance on family level, *p* > 0.05, permutations = 999, model including sequencing depth and the biological covariates, or Linear mixed effect models, q > 0.15). Moreover, we did not observe strong associations between the fecal community types, milk groups (Fig. 3a and b), the season of sample collection or the mode of breastfeeding (exclusive or non-exclusive breastfeeding) with the overall microbiota composition (PERMANOVA, Bray–Curtis distance on family level, *p* > 0.05, permutations = 999, model including sequencing depth and the biological covariates). Models using alternative distance measures, such as Aitchison distances, or performed at the species level, produced comparable results (data not shown). The infant or maternal secretor status, milk group, or any individual HMO did not show any significant associations with the FCTs, or the species alpha diversity or richness (Supplemental Figure 5E and F).

We next assessed the possible associations between the individual HMOs and relative abundances of species-level taxa in the infant microbiota. Significant associations between HMO concentrations and relative abundances of several species were identified such as: negative associations between LSTb and three species of *Klebsiella*, negative associations between LNnT and four species of *Bifidobacterium* and one species of *Bacteroides*, positive associations of 3FL and *B. uniformis* and *P. dorei*, and positive associations between LNDFH2 + 3 and four species of *Bacteroides* as well as with *P. dorei* (Linear mixed effect models, q < 0.15) (Fig. 3c). Strikingly, all the associations’ coefficients between HMO and taxa relative abundances were between −1 and 1, signalling a moderate effect size.

Due to the substantial inter-individual variation of the infant microbiota at 3 months, we next assessed if the associations between HMO composition and the infant microbiota are dependent on the type of gut microbial community. For infants in the FCT1 (n = 88), characterized by a high relative abundance of *Enterobacteriaceae,* and for the FCT2 characterized by a high relative abundance of *Bifidobacteriaceae* and a depletion of *Bacteroidaceae* (n = 56), we did not observe any significant associations. Finally, for samples belonging to the FCT3, characterized by a high relative abundance of *Bifidobacteriaceae* and *Bacteroidaceae* (n = 79), we observed negative associations between *B. thetaiotaomicron* and the concentration of DS-LNT. We also observed a negative association between *P. dorei* and *Bacteroides spp.* and the concentration of LNDFH1. Additionally, *Bacteroides species CAG 443* as well as *Phocaecola vulgatus* were negatively associated to the total neutral component of HMOs. Both *B. thetaiotaomicron* and *Bacteroides species CAG 443* were also negatively associated to the total concentration of HMOs (Linear mixed effect models, q < 0.15) (Supplemental Figure S6).

**Fig. 4:**
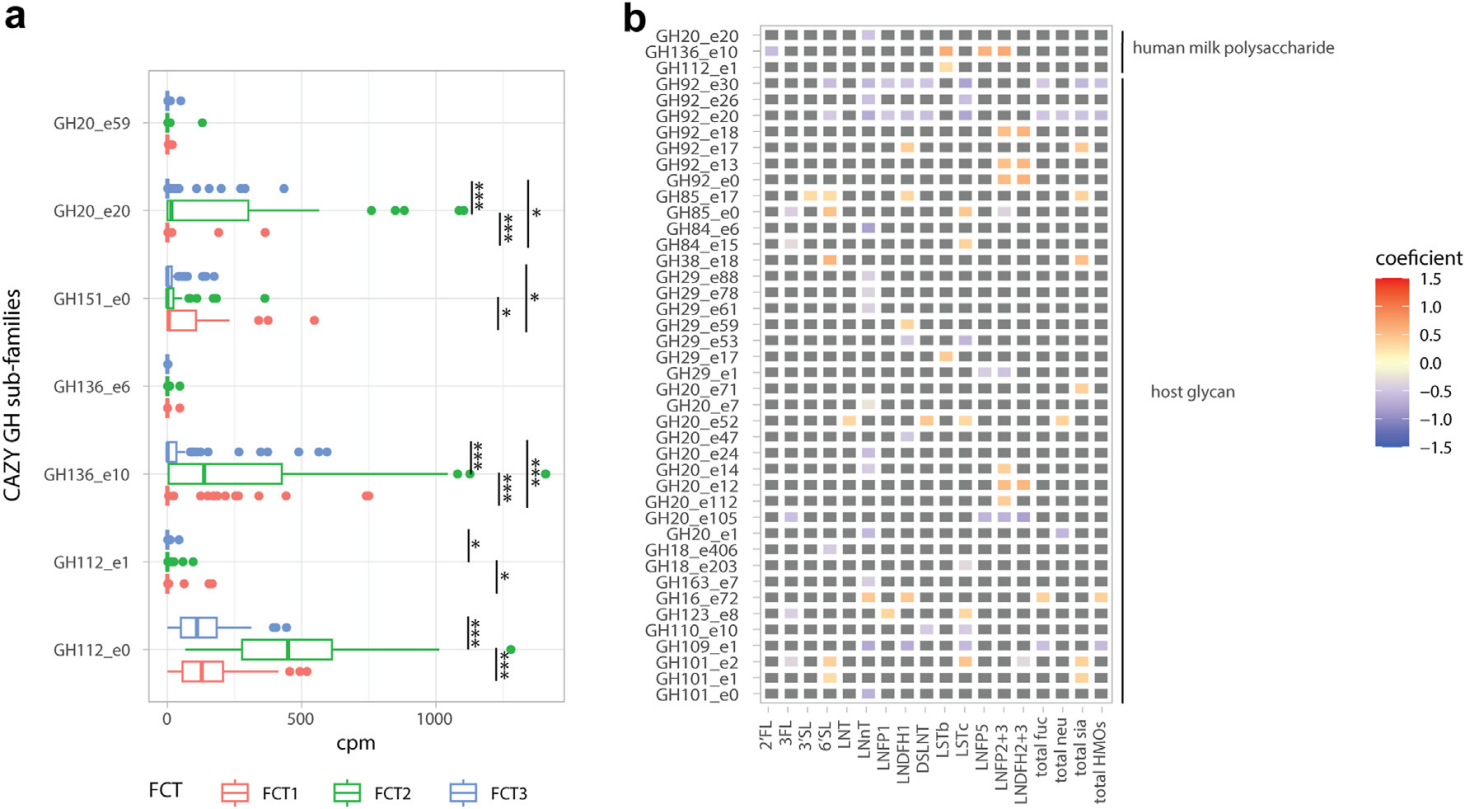
**Association between HMO composition and infant gut microbiota CAZy functional potential at 3 months**. (a) Overview of the infant gut microbiota CAZy functional potential at 3 months for CAZy sub-families involved in HMO degradation. Samples are plotted by FCT, and the CAZy counts in cpm were compared using Wilcoxon test. FDR *p*-values (q-values) are reported as follows: ∗∗∗q-value <0.001, ∗∗q-value <0.01, ∗q-value <0.05. (b) Association between HMO composition and infant gut microbiota functional potential for the total infant cohort at 3 months. Coefficients of association were obtained from linear models on the CAZy sub-families (log transformed gene counts per million reads). Models included parity, birth mode and IA as fixed effects. The associations with FDR adjusted *p*-values (q-values) < 0.15 were considered statistically significant, with original unadjusted *p*-values <0.05. Tile coloring reflects direction and magnitude of coefficient, grey tile represents non-significant association. Only CAZy subfamilies with Human milk oligosaccharides and host glycan as predicted substrate were plotted, however, all significant associations are available in Supplemental File 2.

Finally, we assessed the associations between HMO composition and functional differences in the infant gut microbiota. We investigated the associations between KEGG orthologs and metacyc pathways, and HMOs. However, after adjusting the analysis for biological covariates: birth mode and IA, we did not observe significant associations. We focused our further analysis on carbohydrate metabolism, particularly that mediated by glycoside hydrolases (GHs) involved in the utilization of the HMOs. The three identified FCTs had significantly distinct GH repertoire (PERMANOVA Bray–Curtis distance on GH profiles, *p* = 0.001, permutations = 999, model adjusted for sequencing depth), in particular for the CAZyme sub-families predicted to use HMO as substrates (Wilcoxon, *p* < 0.05) (Fig. 4a). Differences in HMO composition were associated with differential abundances of several GH subfamilies, among which three had HMO as the predicted substrate. Interestingly, a less abundant sialylated HMO, LSTb that was significantly in higher concentrations in secretor mothers' milk, correlated with many CAZy sub-families, however, only with two sub-families predicted to use human milk polysaccharides as substrates (GH112–e1 and GH136–e10). 2′FL and LNnT showed a negative association with GH20–e20 and GH136–e10, respectively (Linear mixed effect models, q < 0.15) (Fig. 4b).

### Association of HMOs with infant gut microbiota differs according to the mode of delivery

**Fig. 5:**
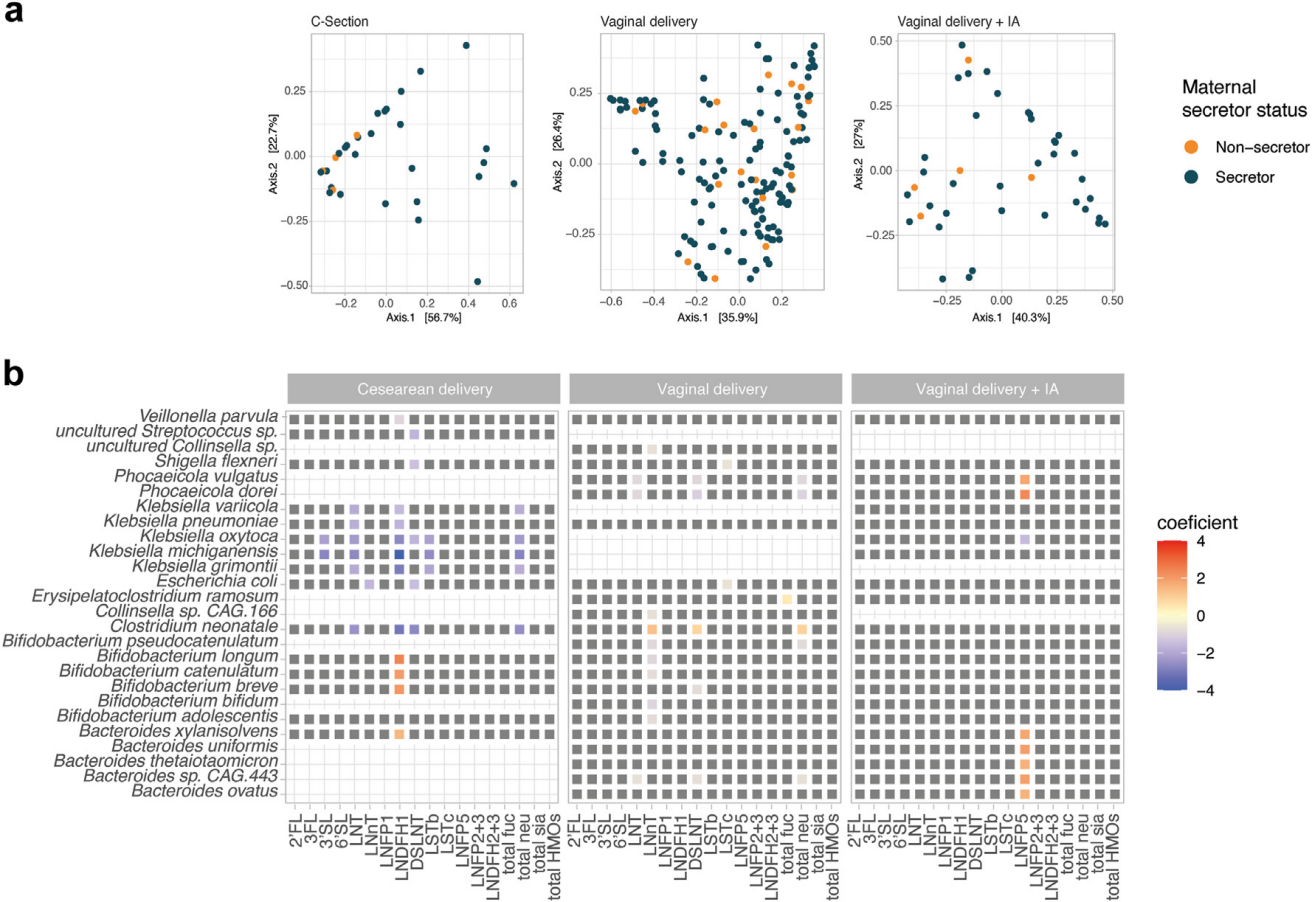
**Association between HMO composition and infant gut microbiota taxonomic composition stratified by delivery mode**. (A) Overview of the infant gut microbiota composition at 3 months colored by maternal secretor status. PCoA on the species-level taxonomic composition of the fecal samples using Bray–Curtis distance stratified by delivery mode. (B) Association between HMO compositions and infant gut microbiota stratified by delivery mode. Coefficients of association were obtained from linear models on the log transformed species-level taxonomic composition, including parity as fixed effects. Only species reaching a minimum relative abundance of 1% in 10% of the stratified sample groups are shown. The associations with FDR adjusted *p*-values (q-values) < 0.15 were considered statistically significant, with original unadjusted *p*-values <0.05. Tile coloring reflects direction and magnitude of coefficient, grey tile represents non-significant association.

As the FCTs were strongly related to the mode of delivery, we further addressed the association between HMOs and infant microbiota at 3 months, now stratifying the samples by delivery mode (vaginally born n = 150, vaginally born with IA n = 41 and with caesarean delivery n = 32). Even with this stratified approach based on delivery mode, no significant association was found between the infant or maternal secretor status and the overall structure of the infant microbiota (Fisher's exact test between FCTs and secretor status, *p* > 0.05 for all strata), or the overall infant microbiota composition (PERMANOVA Bray–Curtis distance on family level, *p* > 0.05, permutations = 999, model adjusted for sequencing depth and parity) (Fig. 5A). Interestingly, in this stratified analysis, as many as 44 statistically significant associations between individual HMO concentrations and the bacterial species in the infant microbiota were identified in a delivery mode specific manner (Linear mixed effect models, q < 0.15) (Supplemental File 2). For vaginally delivered infants, mostly negative associations were observed, in particular between the concentration of LNnT and several *Bifidobacterium* species. However, *Clostridium neonatale* was positively associated to total neutral HMOs. In caesarean delivered infants, a few positive associations involved *Bifidobacterium* species and *B. xylanisolvens* that were associated with LNDFH1, while opportunistic pathogens including *Klebsiella michiganensis, K. grimontii and C. neonatale* were negatively associated to several HMOs, most strongly to LNDFH1. Finally, a positive association between five *Bacteroides* species as well as *Phocaeicola spp.* and LNFP5 was observed in vaginally delivered infants with IA (Fig. 5B, Supplemental File 2). Surprisingly, no additional associations to GH subfamilies degrading HMO could be identified when stratifying the infants by delivery mode and IA exposures (Linear mixed effect models, q < 0.15, Supplemental File 2).

### Association of secretor status with infant microbiota at 6 months and 1 year, and its link with infant phenotypic outcomes until the age of 1 and 2 years

Finally, we assessed the possible associations between secretor status/milk groups and overall infant microbiota at the age of 6 months and 1 year. Since the HMO abundances vary during lactation,^40^ only the effect of maternal secretor status and breastmilk group was assessed here. On the subset of breastfed infants at 6 months (n = 275) and 1 year of age (n = 251), no association was observed on the overall microbiota composition and maternal secretor status or milk groups (PERMANOVA, Bray–Curtis distance on family, *p* > 0.05, permutations = 999, model adjusted for sequencing depth and the biological covariates). These results were consistent when using alternative distance metrics like Aitchison distance, or when aggregating at the genus or at the species level. Similar to the 3-month time point, we analysed the associations between secretor status/milk groups and individual taxa in infant gut microbiota at 6 month and 1 year time points (Linear mixed effect model, q < 0.15), however, we did not observe any associations at the 6 month or 1 year time point either. Furthermore, considering only the subset of breastfed infants at each sampling time point, we also investigated the overall species richness as well as the richness of *Bifidobacterium* and *Bacteroides* species between the children born to secretor and non-secretor mothers, across all birth groups and individually within each birth group (caesarean delivery, vaginal delivery, vaginal delivery with IA). However, we did not find any statistically significant differences (data not shown).We further explored the potential association between maternal secretor status/milk groups, and the following infant phenotypic variables measured at 1 and 2 years of age: diagnosed allergies, BMI z-score, symptoms of atopy, allergy or asthma, overall skin condition reported by the parents, number of antibiotics courses received, and frequency of using gastrointestinal related medications (list of variables in Supplemental File 1). These analyses were conducted both on the total cohort and in subgroups stratified by birth mode and IA. In this cohort, no significant associations were found between maternal secretor status/milk groups and infant phenotypic outcomes in the pooled data or when stratified by birth mode and IA (Fisher's exact test, *p* > 0.05).

## Discussion

In this large-scale study, we investigated the influences of environmental and perinatal factors on the HMO profiles in breastmilk, which in turn showed limited influence on the infant gut microbiota at 3 months of age across the overall dataset. However, interesting birth mode specific patterns were revealed when we studied the associations between HMOs and infant gut microbiota within each birth group: vaginally delivered, vaginally delivered with IA, and caesarean delivered. Also, we did not observe any significant links of either maternal or child secretor status with infant microbiota until the age of 1 year, and infant phenotypic outcomes until the age of 2 years. In our cohort, 86.9% of mothers were *FUT2* secretors, aligning with proportions observed in the Finnish population with 87.2% secretors,^41^ and a multi-country European study with 89.6% secretors.^42^ However, global ratios reported as 80/20 (secretors/non-secretors) indicate substantial variations based on ethnicity.^43^ Non-secretor mothers exhibited significantly higher levels of 3FL, LNFP5, LNFP2 + 3, LNDFH2 + 3, LNT, and LSTb. In contrast, secretor mothers had higher levels of total fucosylated and total HMOs, in particular LNnT, and LSTc. Several studies have previously reported these non-genetically determined HMO variations between maternal *FUT2* secretor status.^6^^,^^11^^,^^23^^,^^24^^,^^44^^,^^45^

The variability in HMO composition and concentrations can arise from biological and environmental influences, quantification platforms, and geographical location among others.^43^^,^^45^ We observed a weak association between the time lapse since the last breastfeeding and sample collection, and higher LSTb values, in a model with milk groups as fixed effects. Such trends were not observed in other studies, partly due to most studies not addressing technical variables. Among the various maternal variables and exposures, after the *FUT2* secretor status, we observed the strongest links between the season of sampling and HMO concentrations. Concentrations of all HMOs except 6′SL, DSLNT, LSTc (all sialylated), and LNDFH2 + 3 varied according to the seasons. The total HMOs as well as the subgroups (neutral, fucosylated, sialylated) were all highest in autumn and lowest in spring, while individual HMOs showed different trends. Seasonality has been reported in a few previous studies. In our study LNnT was highest in summer, while the Canadian CHILD cohort^11^ reported highest LNnT in winter. In our cohort, as well as the CHILD cohort, the highest levels of LNFP2 + 3 (LNFP3 in their case) were found in autumn. Also, other studies from the US and Israel reported marginal seasonal variations in LNFP 3,^44^ LNT, LNFP2, and LNFP3, respectively.^46^ Seasonal variation exists in cow milk oligosaccharides that are up to 10-fold higher in autumn during grazing.^47^ A study on Gambian mothers reported correlations between HMO concentrations and the alternating wet-dry seasons, suggesting associations with nutritional availability.^48^ Also, maternal carbohydrate intake can alter HMO concentrations in breastmilk.^49^ There is seasonal variation in Finland concerning the consumption of vegetables, fruits, berries, and cereals, with fluctuations in folate levels.^50^^,^^51^ However, the link between diet and HMO composition remains unclear, with a few reports suggesting an absence of any associations.^10^^,^^11^ Despite our extensive metadata, we could not investigate the relationship between dietary habits and HMOs due to a lack of sufficient granularity in our dietary data. The variations in HMO concentrations in breast milk might also reflect the influence of other seasonal factors such as exposure to sunlight.

Our results also indicate parity-based variations in HMO composition. We observed that 3FL and 3′SL levels were higher in primiparous mothers in addition to lower LNT levels. Similar to our findings, the CHILD cohort study reported higher levels of LNnT and LNT and lower levels of 3FL in multiparous women.^11^ Several other studies, covering Europe, Asia, and South America, have also reported associations between parity and HMO composition.^6^^,^^10^^,^^42^^,^^45^^,^^52^ The effect of parity has also been observed in the milk oligosaccharide profiles of several animals,^53^ but the mechanisms that explain the relationship are not yet understood. However, we might speculate that maternal immune responses, hormonal changes, as well as differences in mammary gland structure or milk microbiota could have an influence.^54^^,^^55^ Another intriguing, yet unprecedented observation from our study, was the increased levels of LSTc in mothers with IBS. It is important to note that we only had 23 cases of IBS. Moreover, LSTc is a minor sialylated HMO. Further studies with a higher number of IBS cases are warranted to address the potential HMO differences in IBS patients, possibly linked to dietary choices. To the best of our knowledge, there are no previous studies on HMO profiles in IBS mothers. We also observed an association between 6′SL and maternal gestational diabetes, with higher levels of 6′SL, a sialylated HMO, in gestational diabetes. Another study reported elevated levels of a different sialylated HMO, 3′SL, in mothers with gestational diabetes.^56^ It is speculated that higher levels of sialylation during pregnancy is reflective of inflammation, however, the outcomes linked to increased sialylation are still underexplored.^57^ There were a few previously reported associations with HMOs that we did not observe. These include maternal weight, pre-pregnancy BMI, mode of delivery, child sex, and mother's age.^10^^,^^42^^,^^45^^,^^49^^,^^58^ However, it is noteworthy that most of those studies were based on smaller sample sizes.

We explored the relationship between maternal and infant secretor status and infant gut microbiota, until the age of one year. For the 3-month time point, we categorized these samples into fecal community types (FCTs) using hierarchical clustering, revealing three clusters showing strong associations with delivery mode, but not with maternal or infant secretor status. This is counterintuitive, given the fact that the α1, 2-fucosylated oligosaccharides which can only be synthesized by secretor mothers account for a major portion of the HMOs in breast milk in secretor mothers.^40^ Similar findings of limited or weak associations between mother's secretor status/milk groups, and infant microbiota have been reported widely, including in milk collected at earlier time points.^16^^,^^24^^,^^59^, ^60^, ^61^, ^62^ On the other hand, some studies do report differences based on the mother's secretor status in the infant gut microbiota.^23^^,^^63^^,^^64^ Also, it has previously been hypothesised that the secretor status of the infant could have a major effect on the gut ecosystem, since in non-secretor infants the gut mucus is less fucosylated, possibly reducing the attachment sites and nutrients for intestinal microbes.^8^ Similar to the current species-level findings, the broader HELMi cohort (n = 985) showed no association between infant secretor status and genus-level gut microbiota beta diversity.^65^ Interestingly, a metagenomic study including 59 exclusively breastfed US infants reported the infant's, but not mother's secretor status to associate with infant microbiota at 2 months.^63^ Another study investigating infant gut microbiota composition along with mothers' and infants' secretor status found the associations of the latter to be weaker and of shorter term.^66^ In adults, no or weak associations between *FUT2* genotype and gut microbiota composition have been observed.^7^ Based on the current and previous results we can infer that the secretor status of the mother or the infant is not among the major determinants of the infant gut microbiota composition, but specific associations on species-level may occur.

When zooming into the relationship between individual HMOs and infant gut microbiota taxa at 3 months, a striking dependence on the birth mode was observed. We observed a total of 18 associations in the whole unstratified dataset. Even though none of these associations exceeded the effect size of 1, indicating a moderate effect size, the most notable ones were: positive correlations between *Phocaeicola dorei* and 3FL and LNDFH2 + 3, and between several *Bacteroides* species and HMOs such as 3FL, LNFP2 + 3 and LNDFH2 + 3 amongst other weaker correlations with neutral and sialylated HMOs. *Invitro* studies demonstrate the ability of *Bacteroides* spp. to metabolise a wide range of HMOs.^18^, ^19^, ^20^^,^^67^ Also, other studies have shown associations between the abundance of *Bacteroides* and several HMOs, specifically sialylated HMOs.^23^^,^^59^^,^^68^^,^^69^ To the best of our knowledge, previous HMO-infant microbiota studies have not applied stratification by birth mode, while few have used infant community typing.^63^^,^^70^ Upon stratification by fecal community types (FCTs), we observed no associations in FCT1 (characterized by a high relative abundance of *Enterobacteriaceae*) and FCT2 (characterized by a high relative abundance of *Bifidobacteriaceae* and a depletion of *Bacteroidaceae*). However, within FCT3, which was characterized by a high relative abundance of *Bifidobacteriaceae* and *Bacteroidaceae,* we observed several negative associations between HMOs and taxa from *Bacteroides* and *Phocaeicola*. Upon stratification by birth groups, several associations between HMOs and infant taxa were observed. In the caesarean delivered group, we observed negative associations between *Klebsiella* and several HMOs. Positive associations were observed between three *Bifidobacterium* species, and *Bacteroides xylanisolvens* and LNDFH1. In the vaginal group, we observed negative correlations of many taxa with LNnT. In the vaginal + IA group, we observed positive correlations between taxa from genera *Phocaeicola* and *Bacteroides,* and LNFP5. Importantly, we observed a higher number of significant associations between HMO concentrations and infant microbiota composition at 3 months when stratifying the cohort by mode of delivery. This suggests that the effect of HMO composition on the infant gut microbiota may be distinct according to the delivery mode, an insight that can be lost if birth mode is used as a confounder rather than a stratum. Further investigation is required to comprehensively understand the relationship between HMOs and infant gut communities. It is important the mention that considering the naturally low occurring proportions of both caesarean births and non-secretor status, it becomes challenging to discern any patterns with statistical confidence.^71^ We also explored the relationship between glycoside hydrolase (GH) families within the infant gut metagenomes, and HMOs in breastmilk. We found several GH families that exhibited differential abundances between FCTs in relation to HMOs. Upon stratification by birth mode, no additional associations were seen. Notably, only three subfamilies, GH20–e20, GH136–e10, and GH112–e1 were specifically linked to HMO metabolism, with enzymes from the latter family catalyzing the hydrolysis of the β1–3 bond in Lacto-N-biose and the β1–4 bond in N-acetyllactosamine within the primary HMO chain.^72^

We also explored the associations between secretor status and various phenotypic infant variables up to the age of two. Our findings did not reveal any significant associations. Other studies have reported a range of outcomes, such as infant growth parameters, including head circumference, height, and weight,^41^^,^^58^^,^^73^ as well as waist circumference^68^ associating with HMOs. Sprenger and colleagues also reported no differences in BMI or weight between maternal secretor and non-secretor phenotype until the age of four months, which was the duration of their study.^74^ Another report also suggests that HMO concentrations do not significantly impact anthropometric measures and weight gain, implying the nutritional adequacy of breastmilk irrespective of HMO composition.^6^ The absence of any associations between secretor status, microbiota profiles and child phenotypic outcomes, and the absence of strong associations between known HMO consumers such as *Bifidobacterium* spp. and HMOs, may suggest that available glycans, including those independent of *FUT2* and *FUT3*, are sufficient to support healthy infant gut communities and health. It may indicate functional redundancy,^24^^,^^75^ or cooperative interactions among different species in the infant gut.^26^ Moreover, Laursen et al. have highlighted that many bacteria, including *Bifidobacteria*, share machinery for the degradation of a wide range of HMOs, both *FUT2*-related and others.^75^ Interestingly, fecal concentrations of HMOs, instead of breast milk concentrations of HMOs have been reported to strongly associate with the relative abundance of dominant *Bifidobacterium,* and other bacteria in infant feces, suggesting the fraction of undigested HMOs measured from infant fecal samples may be more informative for inferring their utilization patterns by the infant microbiota.^59^ Collectively, this implies that the ingested HMOs in the guts of breastfed infants are in excess, sufficient for supporting infant microbiota, and not completely metabolised. Therefore, it is conceivable that the presence of HMOs plays a more significant role in shaping HMO consumers in the infant gut and supporting infant health than secretor status-based difference or variation between individual HMOs. Furthermore, breastmilk contains various other components that influence infant microbiota, including lysozyme, IgA, and the breastmilk microbiome, all contributing significantly to shaping the infant gut communities.^59^

Our study is a pioneering effort, presenting the largest dataset of its kind comprising 1173 biological samples. With 350 HMO profiles from breastmilk and 823 fecal microbiota profiles from infants until the age of 1 year, we explored links between microbiota, HMOs, maternal secretor status, child secretor status, and child phenotypic outcomes up to age two. Insights into the extensive background and perinatal exposures' impact on HMO profiles are highlighted, along with distinct patterns in microbiota and HMOs for different birth groups and fecal community types. However, we did not investigate the milk microbiota, and due to single timepoint milk sampling at 3 months, temporal changes in HMO profiles were unobserved. It is also important to note that our study was based on a cohort of healthy infants, born in capital region of Finland, which might not be representative of the broader western population. Additionally, we chose a subset of samples from our wider HELMi cohort depending on sample availability and completeness of online questionnaire, suggesting a potential attrition bias. Future research should investigate diet-seasonality interplay, emphasizing underrepresented groups like non-secretors and caesarean delivered infants. Studies including ours that add knowledge to the comprehensive understanding of HMOs, microbiota, and early life exposures have a scope to contribute to advanced strategies for supporting infant health.

## Contributors

DM and AS conceptualized and designed this study. DM, ML, and A-M.M conducted laboratory analyses and measurements of the HMOs. DM and AJP analyzed the data and performed computational experiments. DM, AJP, and AS formulated the study questions, investigated, and interpreted the findings. AJP, D-MG, and AS supervised the study. AS, K-LK, and WMdV designed and established the HELMi cohort. DM wrote the original draft with inputs from AJP and AS. All authors critically revised the manuscript and approved the final version.

## Data sharing statement

The sequence data that support the findings of this study are available under the BioProject ID: PRJEB70237. The names of the repository/repositories and individual samples accession number(s) can be found in the Supplemental File 3.

## Declaration of interests

Authors DM, AJP, AS, K-LK and WMdV are employed by the University of Helsinki and have no conflicts of interest to disclose. Authors ML, A-MM, and D-MG are employees of DSM-firmenich and collaborators via the European Union's H2020-MSCA-ITN-2018 Sweet Crosstalk project.
