# Supplementary material for: Human milk oligosaccharide composition is affected by season and parity and associates with infant gut microbiota in a birth mode dependent manner in a Finnish birth cohort
Source: eBioMedicine. 2024 Jun 4;104:105182. doi: 10.1016/j.ebiom.2024.105182 (PMC11215963; doi:10.1016/j.ebiom.2024.105182)
Supplement: Supplemental Material [file mmc4.docx]

**Supplemental Material**

**Supplemental Table1: Classification of milk groups and secretor status**

**Supplemental Table 2: Characteristics of the study cohort, stratified my mother’s secretor status**

**Supplemental Figure 1: Correlations between HMO concentrations in mother milk**

**Supplemental Figure 2: HMO abundances in mother milk by maternal secretor status**

**Supplemental Figure 3: Average HMO concentrations and Principal Component analysis (PCA) by milk groups**

**Supplemental Figure 4: Heatmaps of taxa and alpha diversity by Fecal community types (FCTs)**

**Supplemental Figure 5: Overview of the infant microbiota, top genera and alpha diversity**

**Supplemental Figure 6: Associations between HMOs and taxa in Fecal community type (FCT) 3**

| HMO | Se+Le+  Milk group 1 | Se-Le+  Milk group 2 | Se+Le-  Milk group 3 | Se-Le-  Milk group 4 |
| --- | --- | --- | --- | --- |
| 2’FL | present | absent | present | absent |
| LNFP1 | present | absent | present | absent |
| LNDFH1 | present | absent | absent | absent |
| LNDFH2 | present | present | absent | absent |

**Supplemental Table1: Classification of milk groups and secretor** status : The milk group and secretor status assignment of breast milk is commonly done based on specific fucosylated HMOs- 2’FL, LNFP1, LNDFH1 and LNDFH2.

**Supplemental Table 2: Characteristics of the study cohort, stratified my mother’s secretor status**
None of the continuous variables in our dataset were normally distributed, they are shown as medians with interquartile ranges (IQR), and the secretor groups were compared using wilcoxon test. Categorical variables are shown in absolute numbers with percentages (%) and significant differences between categorical variables were tested with Fisher’s tests.The p-values of variables that differed significantly between the secretor groups are marked in bold.

A


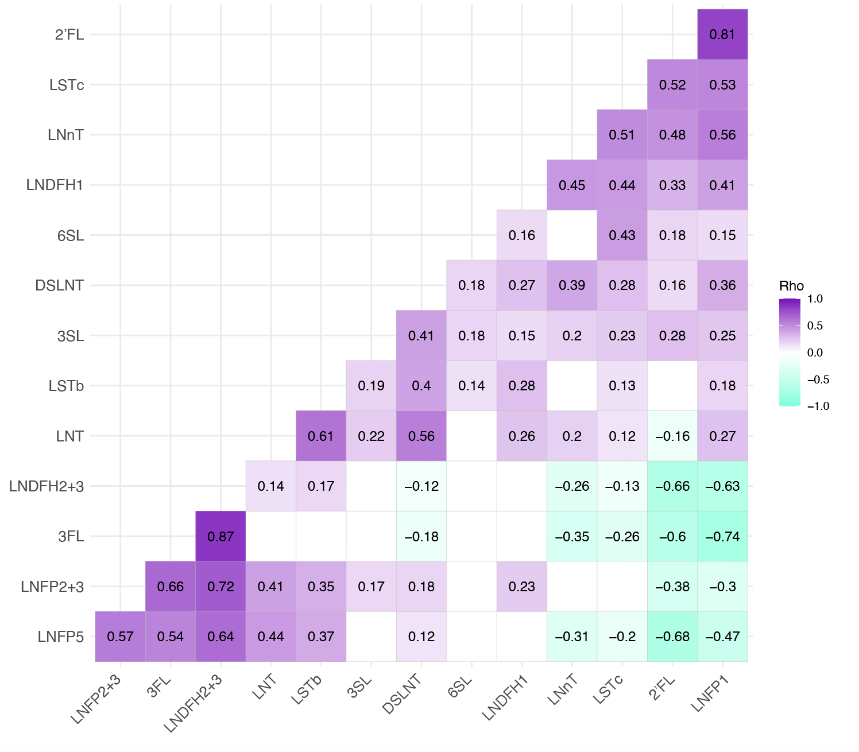


B C


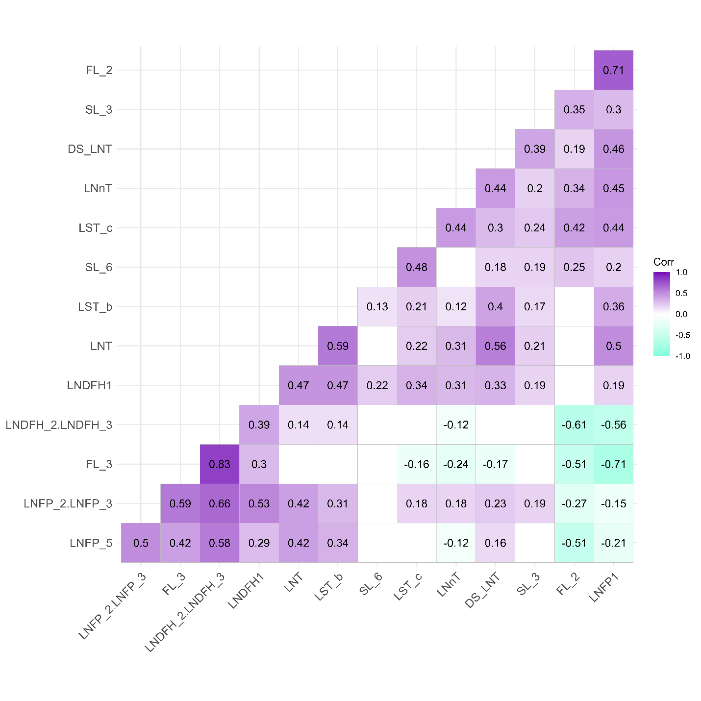

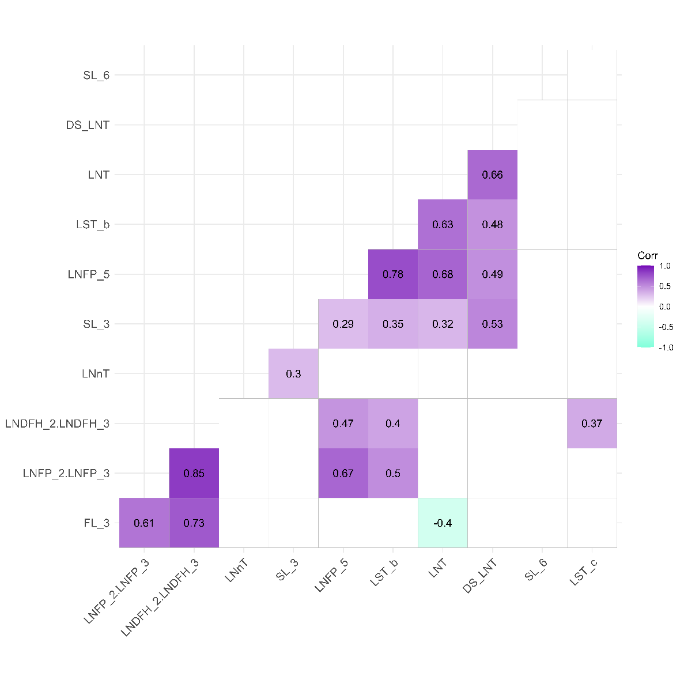


**Supplemental Figure 1: Correlations between HMO concentrations in mother milk**

Spearman’s correlation coefficients between HMOs in (A) the 350 breast milk samples, (B) in secretpr milk samples, and in (C) Non-secretor milk samples. The value in each tile reports the correlation coefficient for significant associations (unadjusted p-value >0.05). The color of the tile indicates the direction and strength of correlation coefficients.


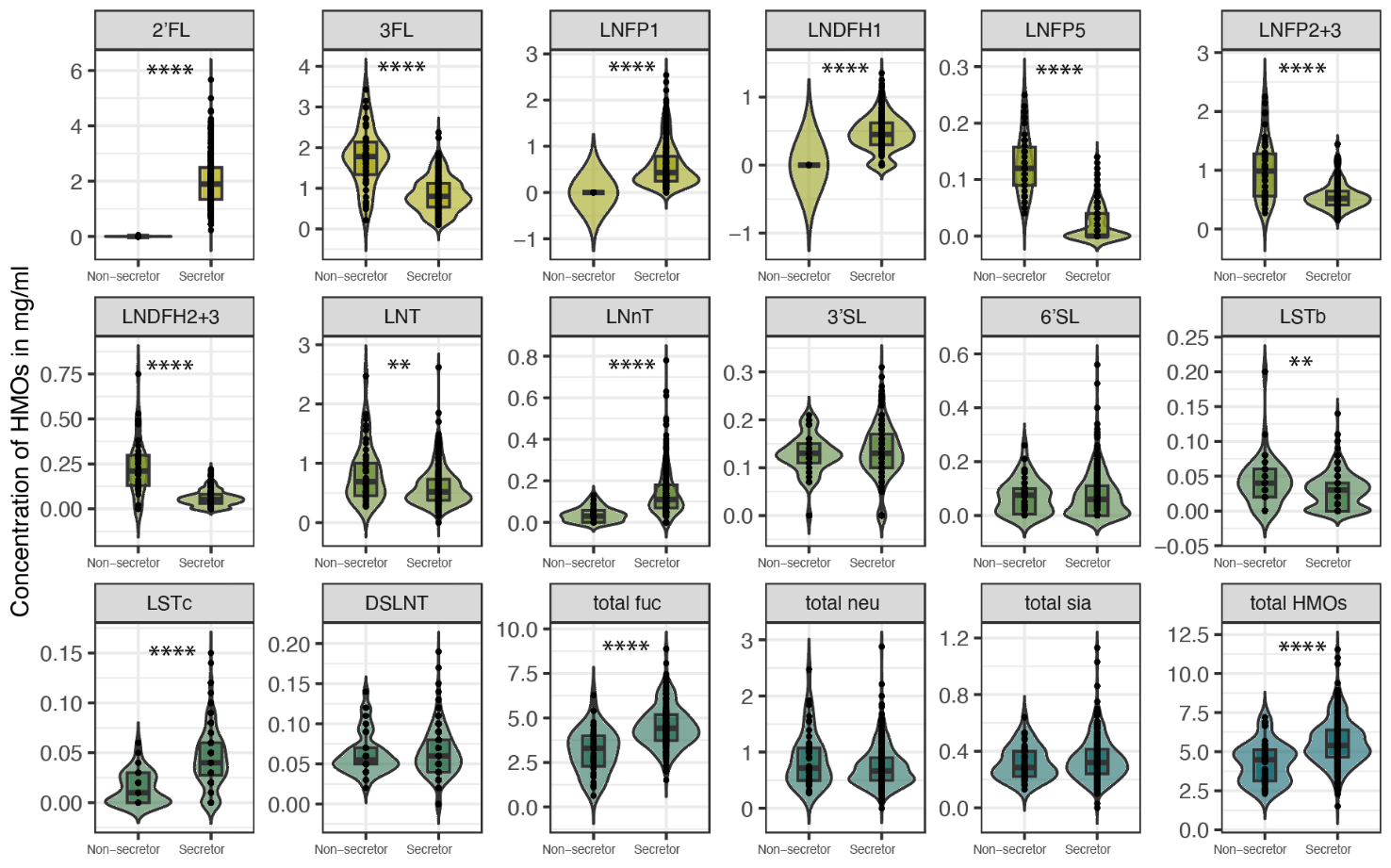

**Supplemental Figure 2: HMO abundances in mother milk by maternal secretor status**

Absolute concentrations of individual HMO and summed class, stratified by Secretor status. Stars denotes significant differences (Wilcoxon test, FDR adjusted p-values (q-values) < 0.05). concentration on y-axes is expressed in mg/ml.


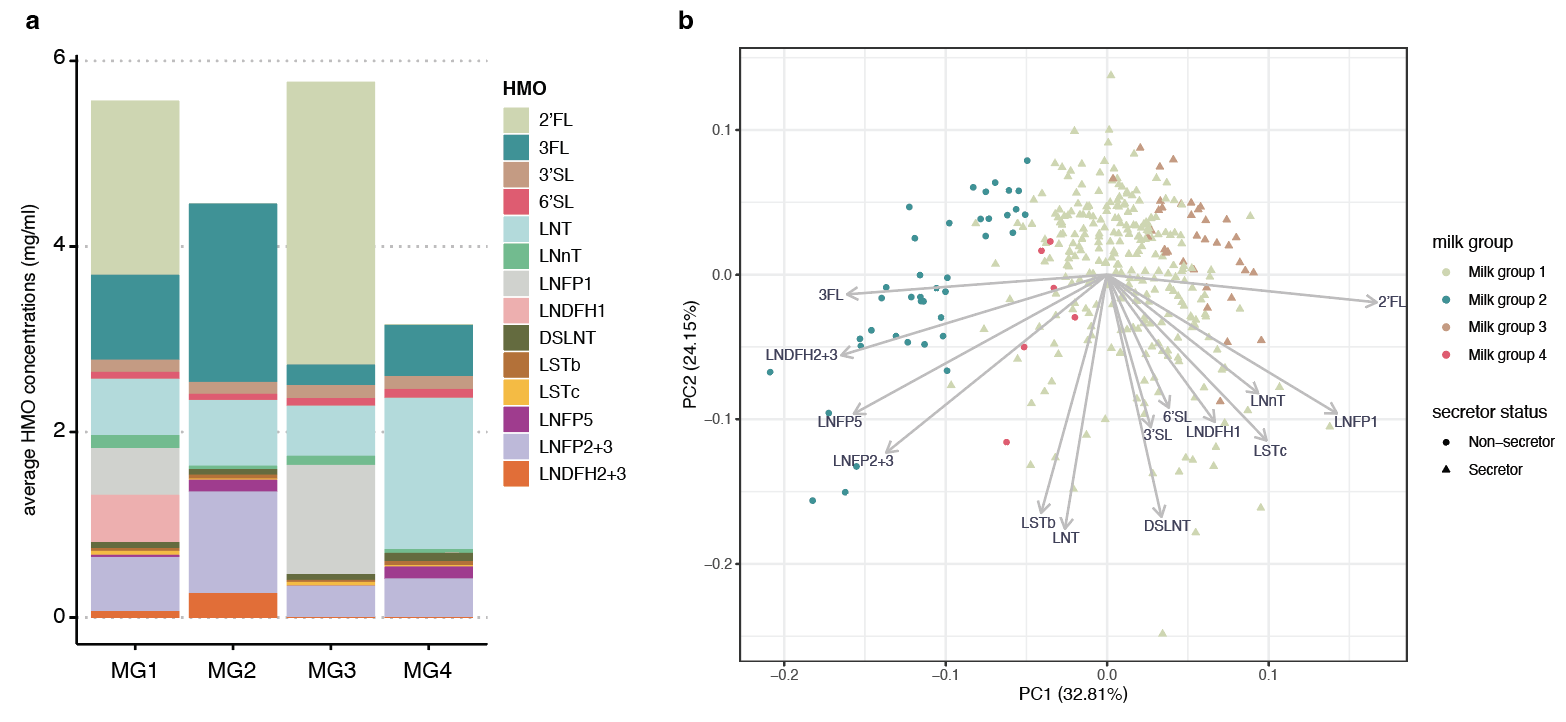


**Supplemental Figure 3: Average HMO concentrations and Principal Component analysis (PCA) by milk groups**

A. Average HMO concentrations in mg/ml by the 4 milk groups (MG: Milk group). B. PCA of the overall HMO composition. The shape of the points denotes mother secretor status, and the color denotes milk groups. The loadings are named with HMOs they are driven by.

a


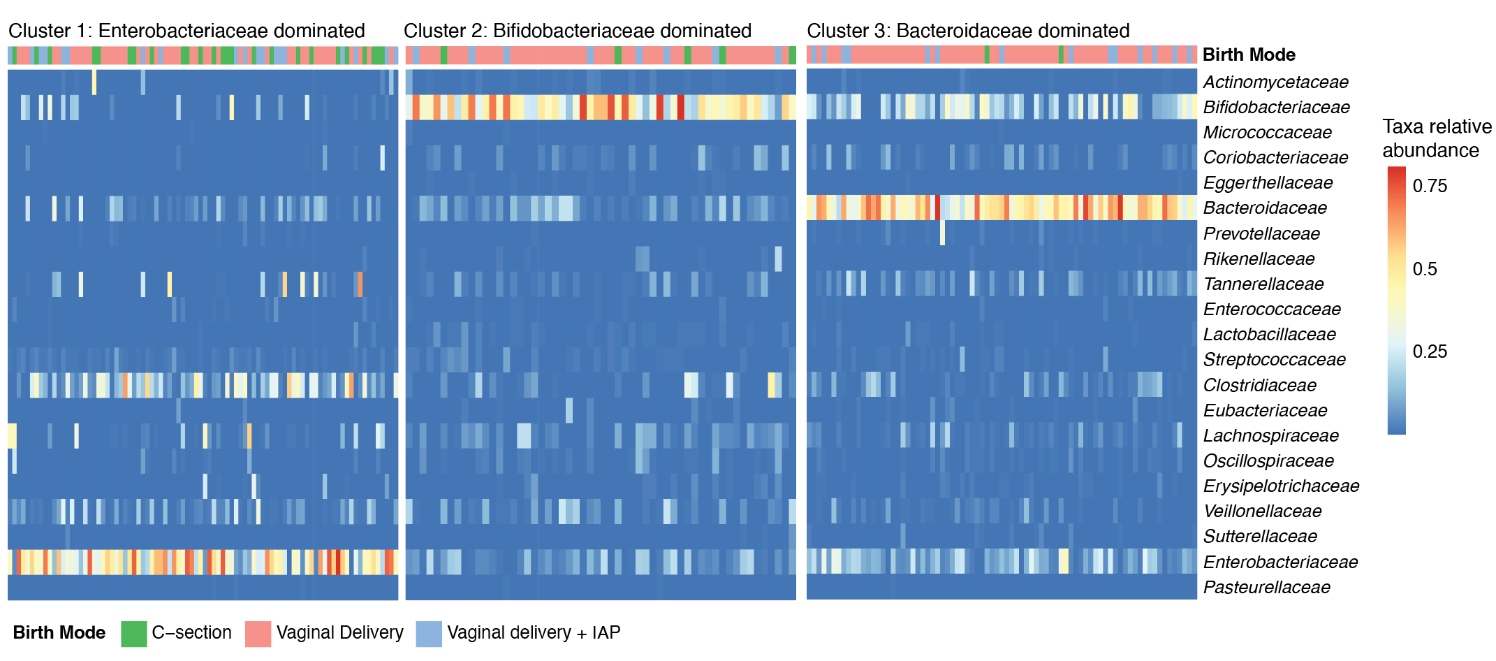


b


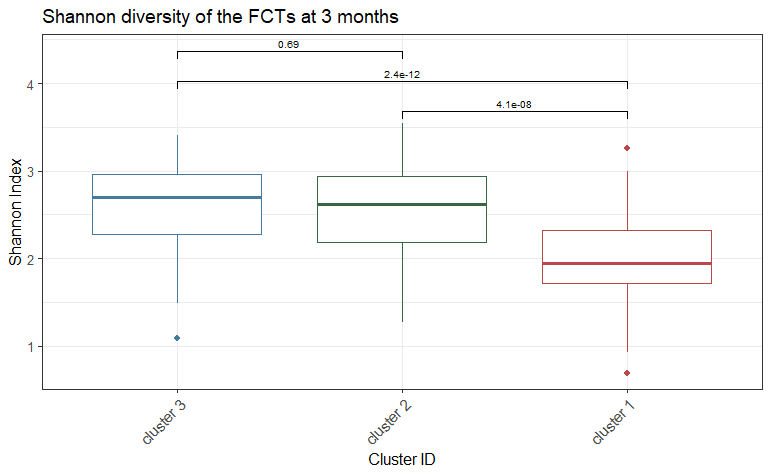


**Supplemental Figure 4: Heatmaps of taxa and alpha diversity by Fecal community types (FCTs)**

**A:** Heatmaps denoting associations between relative abundance of taxa at family level and birth mode within each FCT/cluster. Clusters are labelled by the dominating taxa and tiles are colored by birth mode.

B: Alpha diversity indicated by Shannon’s index at 3 months between the the FCTs/clusters. Differences between the clusters are indicated by p-values shown, which were computed using wilcoxon’s test.

**
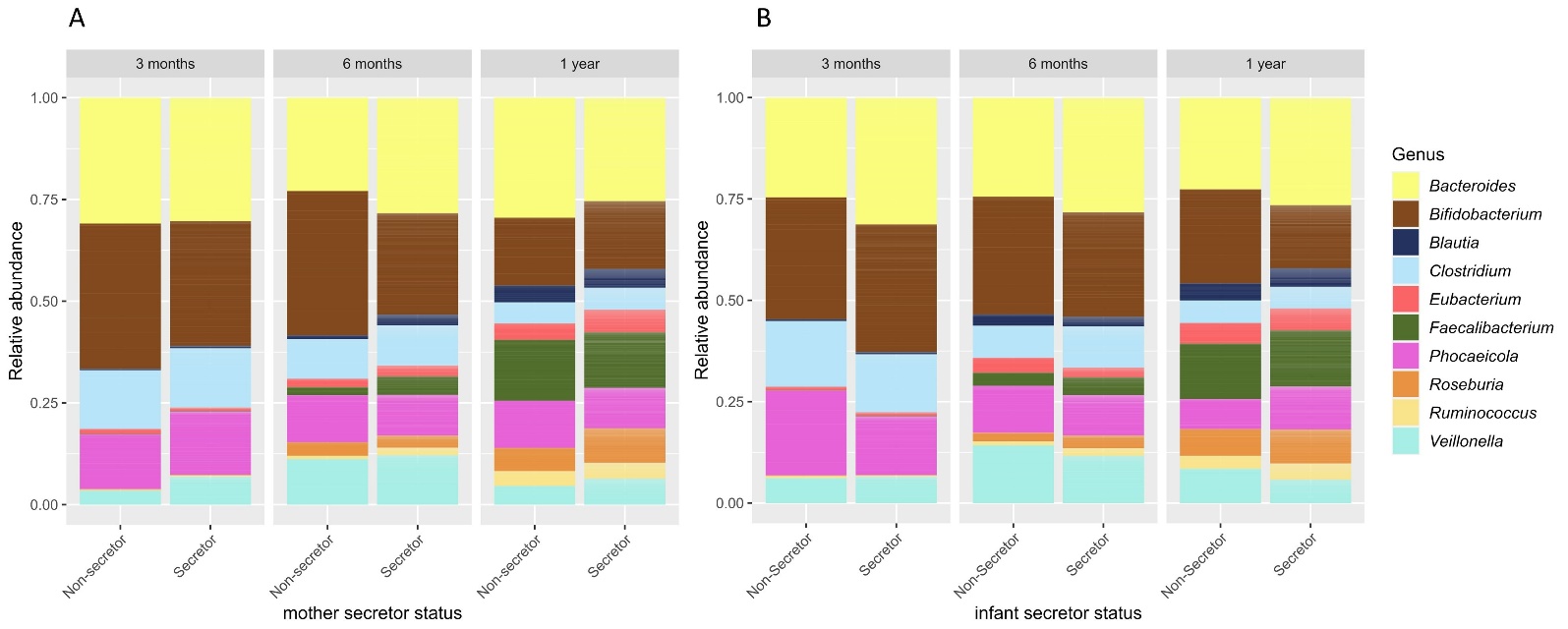
**

**
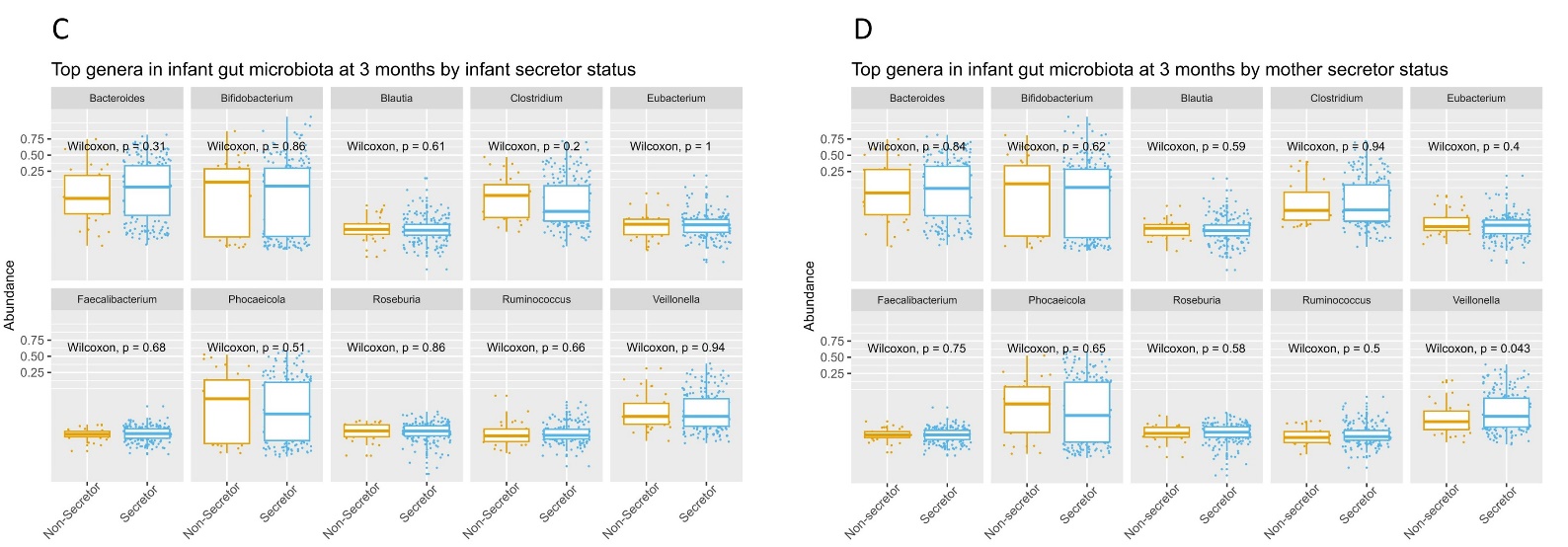
**

**
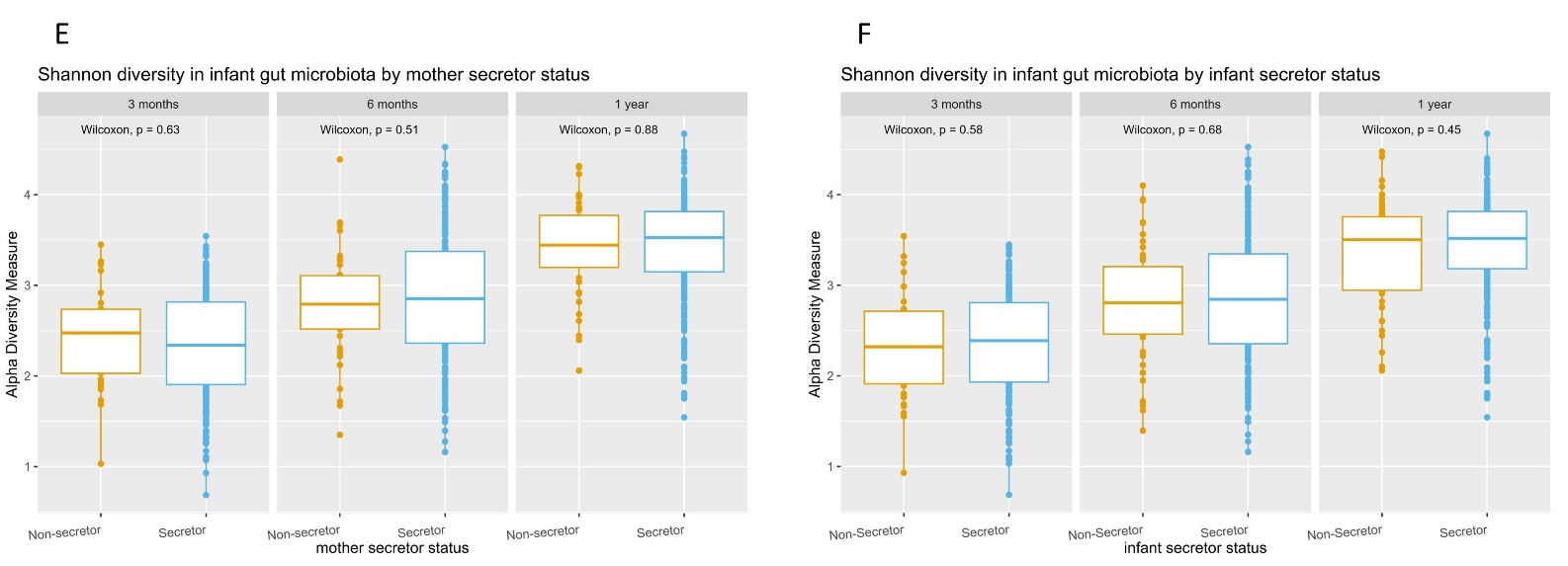
**

**Supplemental Figure 5: Overview of the infant microbiota, top genera and alpha diversity**

A and B: compositional plots of the top genera in infant microbiota at 3,6 and 12 months by mother secretor status (A) and infant secretor status (B)

C and D: top genera in the infant gut microbiota at 3 months by infant secretor status (C) and mother secretor status (D)

E and F: Alpha Diversity indicated by Shannon’s index of the infant gut microbiota at 3 months by mother secretor status (E) and infant secretor status (F)


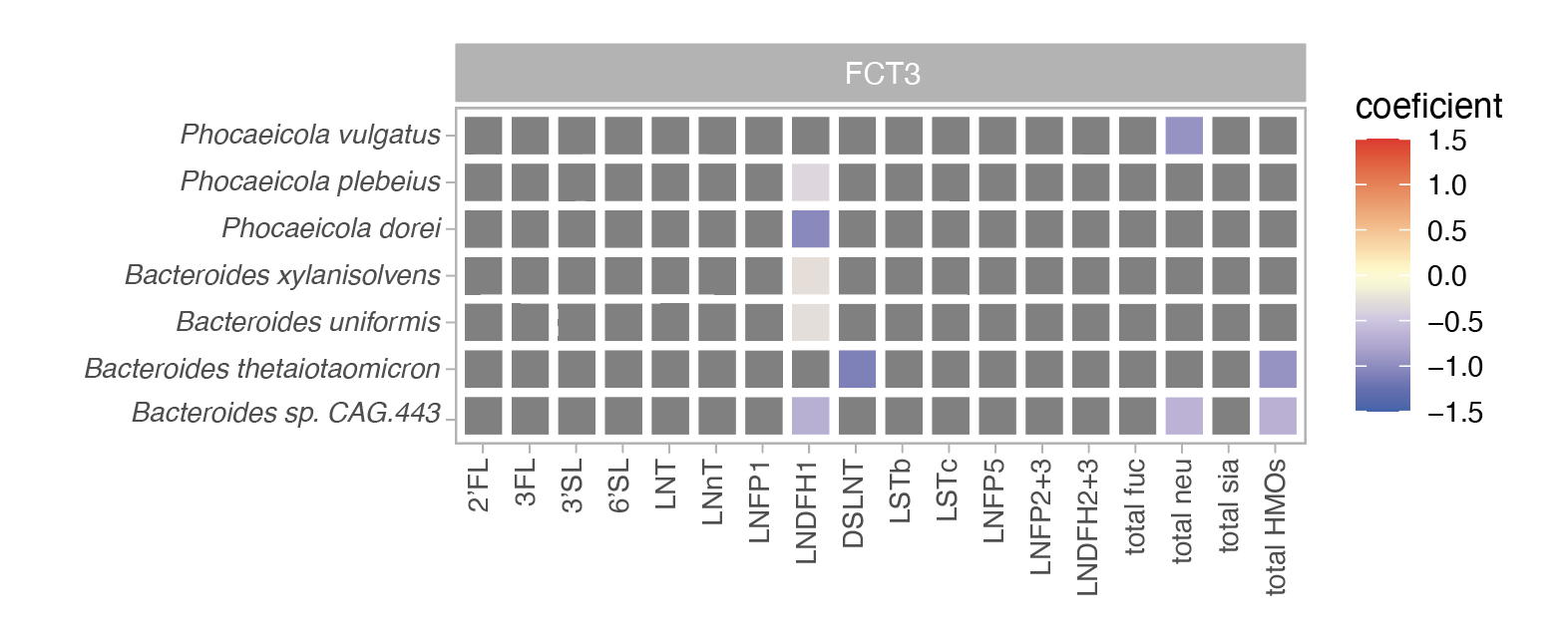


**Supplemental Figure 6: Associations between HMOs and taxa in Fecal community type (FCT) 3**

Association between HMO compositions and infant gut microbiota stratified by FCT. Coefficients of association were obtained from linear models on the log transformed species-level taxonomic composition. Only species reaching a minimum relative abundance of 1% in 10% of the samples in each FCT were considered. The associations with FDR adjusted *q*-values < .15 were considered statistically significant, with original unadjusted *p*-values < .05. Tile coloring reflects direction and magnitude of coefficient, grey tile represents non-significant association.
